# A summary of *Blastocystis* subtypes in North and South America

**DOI:** 10.1186/s13071-019-3641-2

**Published:** 2019-07-29

**Authors:** Paula Andrea Jiménez, Jesús Eduardo Jaimes, Juan David Ramírez

**Affiliations:** 1 Grupo de Investigaciones Microbiológicas-UR (GIMUR), Departamento de Biología, Facultad de Ciencias Naturales y Matemáticas, Universidad del Rosario, Bogotá, Colombia; 2 Escuela de Medicina y Ciencias de la Salud, Universidad del Rosario, Bogotá, Colombia

**Keywords:** *Blastocystis*, Distribution, Diversity, Geographic, North and South America, Subtypes

## Abstract

**Background:**

*Blastocystis* is a stramenopile of worldwide significance due to its capacity to colonize several hosts. Based on its high level of genetic diversity, *Blastocystis* is classified into global ribosomal subtypes (STs). The aim of this study was to conduct a summary of *Blastocystis* STs and depict their distribution throughout North and South America; we did this by assembling maps and identifying its most common *18S* alleles based on diverse studies that had been reported all over the continent and whose *Blastocystis*-positive samples were obtained from numerous hosts.

**Results:**

Thirty-nine articles relating to nine countries from the American continent were considered, revealing that ST1 (33.3%), ST2 (21.9%), ST3 (37.9%), ST4 (1.7%), ST5 (0.4%), ST6 (1.2%), ST7 (1%), ST8 (0.7%), ST9 (0.4%), ST12 (0.3%), Novel ST (1.1%) and Mixed STs (0.2%) occurred in humans. The STs in other animal hosts were ST1 (6.5%), ST2 (6.5%), ST3 (4.7%), ST4 (7.2%), ST5 (15.9%), ST6 (17.3%), ST7 (3.6%), ST8 (20.6%), ST10 (9%), ST14 (3.6%), ST17 (1.1%) and Novel ST (4%). The countries that presented the most abundant variety of studies reporting STs were the USA with 14 STs, Brazil with 9 STs and Colombia with 8 STs. Additionally, new variants had been described in the last few years, which have increased the prevalence of these subtypes in the countries studied, such as Novel ST (1.1%) and Mixed STs (0.2%) in humans and Novel ST (4%) in animals.

**Conclusions:**

This summary updates the epidemiological situation on the distribution of *Blastocystis* STs in North and South America and will augment current knowledge on the prevalence and genetic diversity of this protozoan.

**Electronic supplementary material:**

The online version of this article (10.1186/s13071-019-3641-2) contains supplementary material, which is available to authorized users.

## Background

*Blastocystis*, a strict anaerobic protist, has colonized many different animals around the world and can compromise the gastrointestinal tract of domestic and wild animals such as cattle, dogs, cats, reptiles, birds, chickens and rats, among others [1, 2]. The most probable route of transmission for *Blastocystis* in both humans and animals is *via* oral-fecal transmission, based on the molecular epidemiological data for this parasite. The rapid propagation and the ability to survive in different organisms such as humans and animals, probably explains its global distribution [3].

Several studies have described the genetic diversity present in *Blastocystis*, which has led to its classification as having multiple subtypes (STs) in its different lineages, based on polymorphic regions of its small subunit of the ribosomal RNA gene [4]. Some of these STs are found in different hosts, but others are exclusively in humans [5]. Currently, 17 subtypes are known, of which ST1 to ST9 and ST12 have been identified in humans [6, 7]. In humans from Europe, STs 1, 2, 3 and 4 reportedly occur most commonly [8], whereas ST1, 2 and 3 commonly occur in South America [2, 9]. More than one ST can reportedly colonize humans, and infections with mixed STs have been reported [10].

*Blastocystis* may cause clinical manifestations [11, 12] such as diarrhea, abdominal pain, irritable bowel syndrome, constipation and flatulence [6], along with extraintestinal manifestations such as chronic urticaria [13]. However, these symptoms are not specific from this protist, bearing in mind that polyparasitism is very common in North and South America. It is not known whether these manifestations are associated only with *Blastocystis* and a specific ST, or multiple parasite colonization. However, recent microbiome studies suggest that *Blastocystis* colonization is usually associated with a healthy gut microbiota, rather than with gut dysbiosis generally observed in metabolic or infectious inflammatory diseases of the lower gastrointestinal tract. In addition, a metagenomics approach showed that individuals with intestinal microbiota dominated by *Bacteroides* were much less prone to having *Blastocystis*-positive stool than individuals with *Ruminococcus* and *Prevotella-*driven enterotypes showing that the presence of *Blastocystis* might be beneficial for the human health. The pathogenicity of this organism is under strong debate, mainly due to a high rate of asymptomatic carriers, the differences in host susceptibility, intestinal microbiota and/or different pathogenic potential of different genetic STs [14–20].

Most of the American continent is considered to have ideal conditions (high rates of poverty, inadequate sanitation in poor populations, internal civil conflicts, high biodiversity and lack of potable water in some regions) for a high prevalence of *Blastocystis.* Nevertheless, the *Blastocystis* STs in North and South America are not yet fully understood, given the lack of studies in several of the countries that comprises it. Most is known about its distribution in Colombia, Argentina, the USA, Bolivia, Peru, Brazil and Ecuador [9]. Despite efforts, no consolidation of the metadata has been attempted for the distribution of *Blastocystis* STs and there is only one revision focused on the STs found in humans from South America [9]. Therefore, we describe herein our summary of the studies published on *Blastocystis* subtypes in humans and other animals across North and South America. We constructed maps for *Blastocystis* and were able to identify its most frequent *18S* alleles.

## Methods

### Literature searches

We searched for articles reporting on the presence of *Blastocystis* STs in humans and other animals throughout North and South America in the following databases: PubMed, ScienceDirect and the Integrated Search System of Universidad del Rosario, Colombia. The included keywords were *Blastocystis*, STs, subtypes, distribution, epidemiology, alleles, molecular, geographic, intestinal parasites, genetic diversity and characterization.

Studies reported in English, Portuguese and Spanish were selected. We geographically limited our searches to studies from North and South America, excluding those that were undertaken outside of the American continent. The information on the articles included their publication dates, summaries and results, and whether the *Blastocystis* subtype and study system (model animal or human) was mentioned. The inclusion criteria were as follows: articles from which samples were obtained in countries on the American continent, identification of the parasite by one or both parasitological and molecular methods, and *Blastocystis* subtype analysis. Taking the above into account, approximately 50 articles were found, of which only 39 met the above-stated criteria to be part of this review.

### Information extraction

Two investigators performed data extraction during January and February of 2019; extracting the characteristics of each study, which included the country, exact location of the samples, number of samples, number of samples positive for *Blastocystis*, host, subtype identification, number of samples per subtype, alleles identified, method used for subtype identification, last name of the first author and year of publication. A Microsoft Excel database was constructed with the information obtained from the articles, in which all the data above-mentioned and variables were added, to tabulate the information in an efficient way (Additional file 1: Table S1). We extracted the information on the variables from each of the articles that met the inclusion criteria for this study. This information was supplemented by searching for the coordinates (latitude and longitude) of the different places where the samples were collected. Thus, the data obtained were built with the QGIS maps program, thereby revealing the distribution of STs in North and South America and the ST variables, the country and the geographical region, with their exact coordinates, which allowed us to locate the specific geographical points for the STs. This was done for the STs that are most prevalent in both humans and animals (ST1, ST2 and ST3). Finally, a map of North and South America was constructed in which all the subtypes of the STs found in this study and their presence in the different countries were taken into account, which allowed us to identify which subtypes occurred in the nine countries that had carried out typing studies.

## Results

**Fig. 1:**
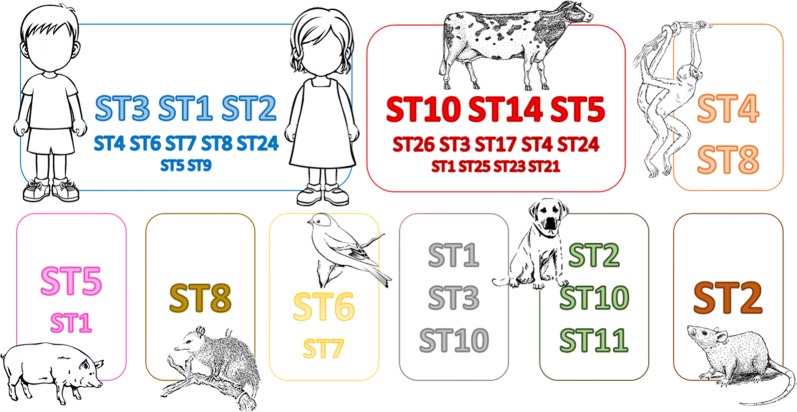
*Blastocystis* subtypes found in humans and animals. The boxes show the *Blastocystis* subtypes per host, and the size of the number is proportional to parasite occurrence. There are vast differences in the prevalence between these as groups for instance. This figure depicts occurrence and not prevalence

Our review of *Blastocystis* in the different countries of North and South America identified 39 articles that met the selection criteria, for which the distribution of *Blastocystis* and its subtypes was analyzable. However, only nine countries in North and South America (Argentina [9, 13], Brazil [9, 11, 21–31], Bolivia [9, 32, 33], Colombia [2, 9, 10, 34–37], Chile [38], Ecuador [9, 39], USA [17, 36, 40–47], Peru [9] and Mexico [48–50] were found to have carried out this type of study. From these countries, *Blastocystis* was identified in samples from both human and other hosts. *Blastocystis*-positive samples were recorded for birds (*Gallus gallus domesticus*) [2, 28], pigs (*Sus scrofa domestica*) [26, 36, 41], monkeys (*Alouatta* spp.) [2, 39], marsupials (*Didelphis marsupialis*) [2], cattle [2, 26, 36, 41, 44], cats (*Felis silvestris catus*) [26, 45], dogs (*Canis lupus familiaris*) [2, 23, 26, 27, 45], sheep (*Ovis orientalis aries*) [26] and rats (*Rattus rattus*) [2]. The STs found by host (human or animal) are shown in Fig. 1.

### Summary of *Blastocystis* STs by country

**Fig. 2:**
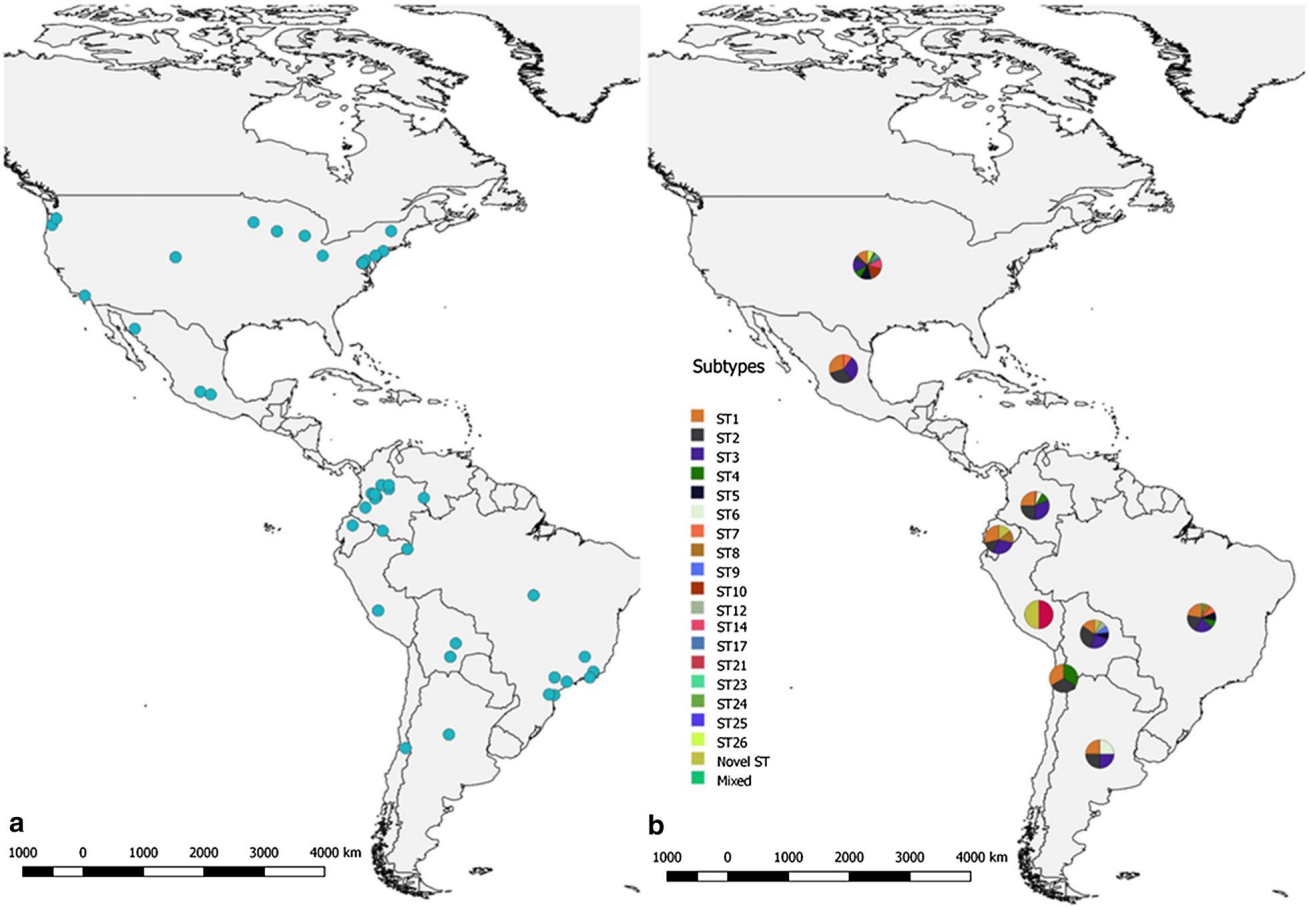
**a** Distribution of *Blastocystis* in North and South America based on the positive sample reports by country. **b** Distribution of *Blastocystis* subtypes in the different countries where samples have been typed

**Fig. 3:**
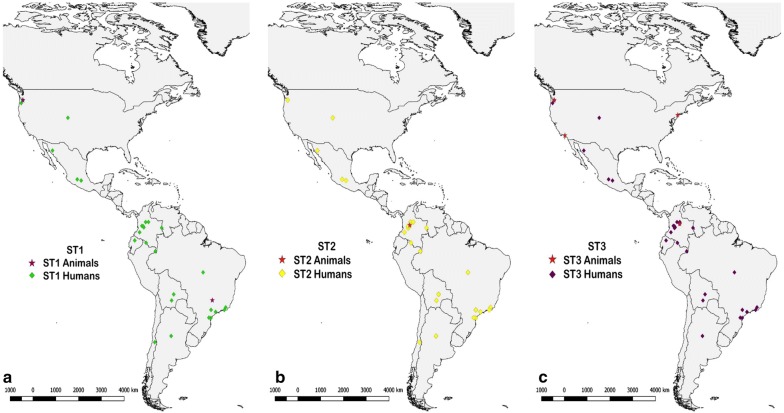
**a** Distribution by country of the samples positive for ST1 in humans and other animals. **b** Distribution by country of the samples positive for ST2 in humans and other animals. **c** Distribution by country of the samples positive for ST3 in humans and other animals. *Abbreviation*: ST, ribosomal subtype

The *Blastocystis* distribution throughout North and South America, based on the studies conducted thus far in 9 different countries (USA, Mexico, Colombia, Brazil, Ecuador, Peru, Bolivia, Chile and Argentina), is shown in Fig. 2a, and the composition of the subtype categories is shown in Fig. 2b. The most frequent subtypes (ST1, ST2 and ST3) that were identified in humans and other animals are shown in Fig. 3.

Among the studies conducted in North and South America, the most widespread subtypes we collated were ST1 and ST2, which were present in the samples from eight of the nine countries that were studied [2, 9–11, 17, 21–27, 29–39, 42, 43, 45–50]. Subsequently, ST3 was found in seven of the nine countries [2, 9–11, 13, 17, 21, 23–25, 27, 30, 31, 33–37, 39, 42, 43, 46–50]. To a lesser extent, Novel ST was present in five of the nine countries [9, 39], ST4 and ST8 occurred in four countries [2, 9–11, 21, 22, 25, 30, 31, 35–39, 42, 44, 47], ST6 and ST7 occurred in three countries [2, 9–11, 13, 23, 27, 28, 30, 35, 50], ST5 occurred in two countries [9, 36, 41, 44], while ST9, ST10, ST12 and ST17 occurred in only one of the nine countries that were studied [9, 32, 40, 44, 45].

The high occurrence of ST1 led us to build a map (Fig. 3a) georeferencing the *Blastocystis*-positive sampling sites by country. The map in Fig. 3a shows the ST1-positive samples obtained from humans, which are represented by green points. This ST was identified in studies from Argentina, Bolivia, Brazil, Chile, Colombia, Ecuador, USA and Mexico [2, 9, 11, 13, 21–25, 27, 29–35, 37–39, 42, 43, 46–50]. In addition, positive samples for ST2 shown in panel b of the map in Fig. 3 (represented by yellow points) are limited to Argentina [13], Brazil [9, 11, 21, 22, 24, 27, 29–31], Bolivia [9, 32, 33], Colombia [2, 9, 35, 37], Ecuador [39], Chile [38], USA [38, 46] and México [49, 50]. The ST3 positive samples, represented in Fig. 3c (violet points), were distributed in Argentina [9, 13], Brazil [9, 11, 21–25, 27, 30, 31], Bolivia [9, 33], Colombia, [2, 9, 34, 35, 37], Ecuador [9, 39], USA [42, 43, 46, 47] and Mexico [48–50].

Likewise, the georeferenced distributions for other hosts are also represented in Fig. 3, where ST1 samples (purple stars) were obtained from Brazilian pigs [26], Colombian cattle [2] and from North American dogs and cats [45]. For ST2 shown in panel b of the map (Fig. 3b) by red stars, the samples were recorded from Colombian dogs and rats [2]. Moreover, ST3 samples, represented in Fig. 3c by pink stars, were limited to Colombian departments [2] and the USA [45].

### Distribution of *Blastocystis* STs by hosts

The STs distribution for humans was as follows: ST1 (615/1847; 33.3%), ST2 (404/1847; 21.9%), ST3 (700/1847; 37.9%), ST4 (31/1847; 1.7%), ST5 (8/1847; 0.4%), ST6 (23/1847; 1.2%), ST7 (18/1847; 1%), ST8 (13/1847; 0.7%), ST9 (8/1847; 0.4%), ST12 (4/1847; 0.3%), Novel ST (20/1847; 1.1%) and Mixed STs (3/1847; 0.2%) (Fig. 2b). In the case of the non-human animals, the distribution was as follows: ST1 (18/267; 6.7%), ST2 (18/267; 6.7%), ST3 (13/267; 4.9%), ST4 (20/267; 7.5%), ST5 (44/267; 16.5%), ST6 (48/267; 18%), ST7 (1/267; 0.4%), ST8 (56/267; 21%), ST10 (25/267; 9.4%), ST14 (10/267; 3.7%), ST17 (3/267; 1.1%) and Novel ST (11/267; 4.1%).

**Fig. 4:**
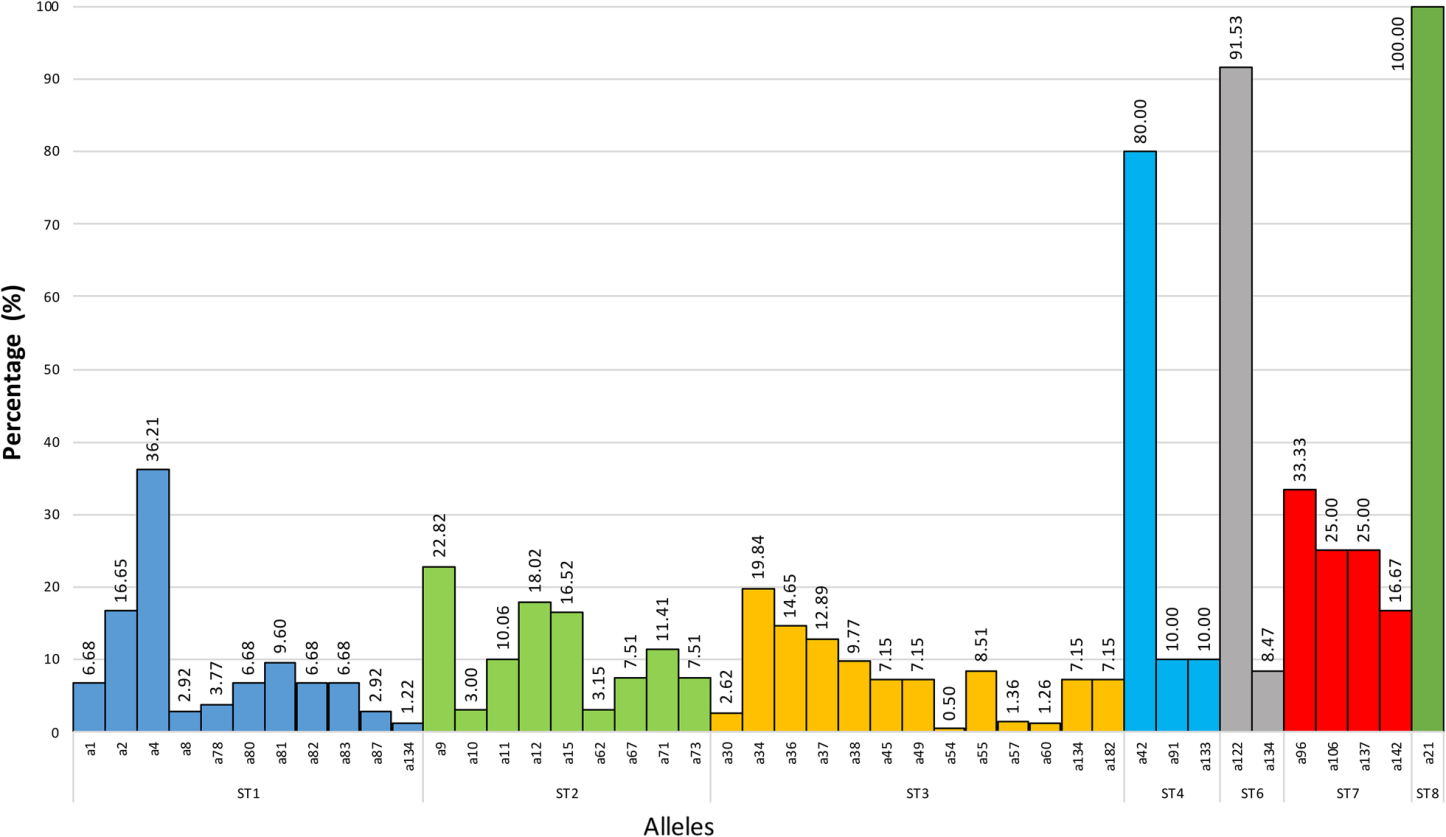
Distribution of *18S* alleles in *Blastocystis* based on the positive samples for each subtype in humans

**Fig. 5:**
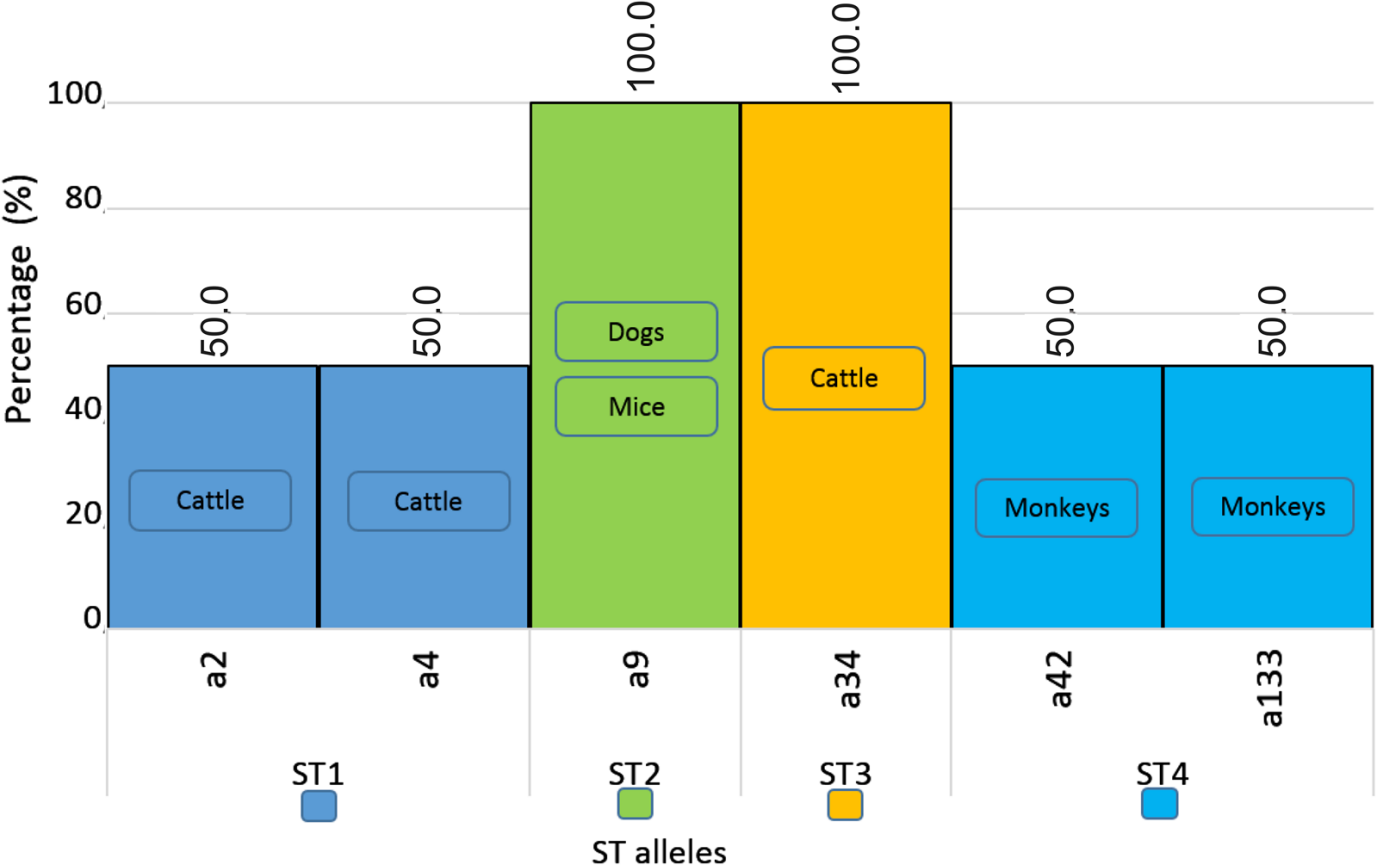
Distribution of *18S* alleles in *Blastocystis* based on the positive samples for each subtype in cattle, dogs, mice and monkeys

According to the allelic discrimination relating to the identified subtypes in humans, the most frequent alleles in each ST were as follows: ST1 (a4, 2) also found in American cattle [2, 10, 11, 23, 30, 35, 46], ST2 (a9, 12, 15, 11, 71) where a9 was also present in dogs an rats [2, 10, 11, 23, 27, 30, 33, 37, 46], ST3 (a34, 36, 37) with a34 also in American cattle [2, 9, 11, 13, 23, 27, 30, 35, 37, 46], ST4 (a42, a91, a133) where a42 and a133 were found in *Alouatta* spp. [2, 10, 30, 35, 37], ST6 (a122) also described in chickens [2, 10, 11, 23, 30], ST7 (a96, 106, 137, 142) [10, 23, 27] and ST8 (a21) and *Didelphis marsupialis* as well [2, 30]. The shared alleles led us to hypothesize that they can have an important role in the transmission dynamics of the parasite between different hosts, but this subject is not analyzed in the present study. Additionally, other alleles were identified in smaller amounts (≤ 10%) in the STs mentioned: for ST1, other 9 alleles were found; ST2, 4 alleles; ST3, 10 alleles; ST4, 2 alleles; and ST6, 1 allele. This information was obtained from those studies (*n* = 10) in which allelic detection was carried out; however, not all of them used this methodology, so no alleles were identified in some of them. This means that the information shown in Figs. 4 and 5 correspond only to those studies that performed allele typing of the samples obtained in humans and other animals, respectively.

## Discussion

The most recent epidemiological data on the *Blastocystis* STs in North and South America are limited to reports from specific countries such as the USA, Colombia and Brazil, where the majority of reports originate [9, 10, 29–31, 35, 44]. This prompted us to conduct this review whereby we considered every country in North and South America where *Blastocystis* has been studied; however, data are not available for many countries. The fact that the majority of reports come from these countries may be related to their higher numbers of investigators. This suggests that more investigators are required in the underrepresented countries so that the true distribution of *Blastocystis* across North and South America can be elicited.

Considering the above information, this review found that STs 1 to 9 were present in the samples from the North and South American countries that have studied and typed *Blastocystis*. Although the literature mentions that these STs only colonize humans (Additional file 1: Table S1) [5], this review revealed the presence of these subtypes in other animals such as monkeys (*Alouatta* spp.), pigs, birds, cats, cattle, dogs, marsupials (*D. marsupialis*) and rats (Fig. 1). This suggests that these animals might be in contact with humans, either domestically or as farmed animals, making it possible for them to be colonized by an ST that was thought to occur only in humans, as is considered the case with ST3 [21]. This highlights the zoonotic potential of this stramenopile and its ability to colonize different host species, an observation widely reported in different countries in Europe, Africa and Asia [3, 6, 51–59]. This is supported by the *18S* allele data where multiple alleles are shared between humans and animals.

The present review found that a large number of samples were typed most frequently as ST1, ST2 and ST3 in humans, followed by other STs in minor percentages, with values of 33, 22, 38 and 7%, respectively (Fig. 3), in agreement with the values reported in a previous study [9]. The countries included in this review had shown in Argentina the highest number of samples were positive for ST3 (Figs. 2, 3) [13], Bolivia showed the presence of ST9 and ST12, being the only country that presents these subtypes in North and South America. The USA was the only country that detected ST5 [33, 44]. Brazil had a highest prevalence of ST7, but this subtype was also identified in Mexican and Colombian samples [9, 10, 23, 27, 28, 50]. Interestingly, Colombia is one of the countries where a greater variety of genetic variation is seen, and where the presence of almost all subtypes was found.

ST8 has only been found in marsupials (*D. marsupialis*) and ST6 was found in both in humans and birds in Brazil [9, 10, 23, 27, 50]. In Chile, ST1, ST2 and ST4 were identified, although studies in this country have only been conducted in humans [38]. One of the few countries where ST8 was detected is Ecuador, in *Alouatta* monkeys [39]. Of note, the USA reports on a genetic variant known as ‘Novel ST’ (ST21, 23–26) and is the only country where ST14 and 17 were found in cattle [36, 40, 44]. In Mexico ST1, ST2 and ST7 were identified in *Blastocystis*-positive samples (Fig. 2) [9, 50].

In other parts of the world such as Europe, the most abundant STs recorded were ST3 and ST4 [8, 60], and the present study identified these subtypes at prevalences of 38% and 1.7%, respectively, although there is disagreement about ST4, which in our analysis was the fourth most common subtype. The possibility exists that the ST3 is associated with transmission in humans because of the large quantity of positive samples and because the infections reported result from human-to-human transmission; nonetheless, ST3 has spread in non-human species that are in contact with people (e.g. cats and cattle) [2, 44, 45]. Therefore, the hypothesis of ST3 being of human origin is not supported by the present study, but it is the most abundant subtype in humans from North and South America.

Although previous studies reported that ST4 is only present in Colombia, it has since been identified in Brazil, Chile and the USA, suggesting that the patterns of transmission for *Blastocystis* have allowed it to spread geographically, and also that there is insufficient documentation on the presence of this subtype in North and South America [30, 35, 42]. According to the hypothesis of Ramirez et al. [9], ST4 is considered a minor infection in continental American animals because of the specific pathogen-host interactions on this continent or genetic characteristics as not yet known, in addition to the lack of studies on this premise. As the samples from which this subtype arose were from Colombia, Brazil, Chile and the USA, it is possible that this ST was carried by migration from the European continent to America by infected individuals who had visited these countries. This might explain the slight increase in infected individuals observed in the present study over previous studies, and it might also explain the appearance of this subtype in new countries where it has not been reported before, such as Brazil, Chile and the USA. Furthermore, its occurrence rates in animals such as monkeys (*Alouatta* spp.) and cattle in the actual studies under review were based on very few samples, making it difficult to establish reliable associations about ST4 and its hosts. However, it cannot be ruled out that these host animals may have some degree of genetic susceptibility to ST4 infections or might even have had their infections transmitted to them by infected humans. It would be interesting to establish whether the microbiome composition of the host animals might influence which subtypes infects them.

As for Novel ST, our review found that it is not present across North and South American countries, but studies on this ST have been performed in the USA to determine whether genetic differences exist between samples that fall within this ST. Hence, the term ‘Novel ST’ is now being replaced by numbers that follow ST17 by some authors, and this new numbering applies to cattle in the different states of the USA. Specifically, ST26 was the most frequently found ST in four US states (Michigan, New York, Washington and Wisconsin) and ST24 was found in two states (California, New York) (Fig. 2) [44]. This indicates that it is necessary to conduct further studies to corroborate the genetic diversity in the newly emerging subtypes at the molecular level and investigate whether they are present in other species and in other countries. Clearly, it is now important to harmonize the current nomenclature used in this field because some STs have been reported using different regions of the *18S* gene and not the consensus one reported by Scicluna et al. [60]. Concerningly, the entire *18S* has not been sequenced to demonstrate they are true novel STs. Future studies should consider sequencing the entire *18S* in order to place them as truly new STs or just variants of the currently known STs. In fact, we sequenced the entire *18S* region of those called “Novel STs” reported by Ramírez et al. [9] and found these were variants of ST6 and ST8.

## Conclusions

In recent years, a variety of molecular epidemiological studies have been conducted on *Blastocystis* to identify its subtypes in the different countries from North and South America, but there is still too little data to elucidate the circulating subtypes and ribosomal alleles in these regions. It is important to highlight that the vast biodiversity on this continent could be shaping the emergence of new STs. We encourage the scientific community to commence subtyping this protist in several domestic and wild animals to obtain a better picture of *Blastocystis* in the region. We critically suggest that action should be taken regarding whether the new subtypes reported are in fact new subtypes or just variants, sequence artefacts, etc. Scientists in the *Blastocystis* community share the responsibility to not confuse and mix-up *Blastocystis* terminology. We finally call for action from researchers working on intestinal parasitism to start depicting the *Blastocystis* STs across the whole American continent (mainly Central American countries where information is lacking) to complement the maps and STs distribution presented herein.

## Additional file

**Additional file 1: Table S1.** Microsoft Excel database assembly of all the information from the articles included in this review.

## Data Availability

The dataset used in the present review is summarized in Additional file 1: Table S1.

## Abbreviations

OTU: operational taxonomic units
STs: ribosomal subtypes
